# TNF induces pathogenic mitochondrial ROS in tuberculosis through reverse electron transport

**DOI:** 10.1126/science.abh2841

**Published:** 2022-06-24

**Authors:** Francisco J. Roca, Laura J. Whitworth, Hiran A. Prag, Michael P. Murphy, Lalita Ramakrishnan

**Affiliations:** 1 Molecular Immunity Unit, Cambridge Institute of Therapeutic Immunology and Infectious Diseases, Department of Medicine, University of Cambridge, Cambridge CB2 0AW, UK; 2 MRC Laboratory of Molecular Biology, Cambridge CB2 0QH, UK; 3 MRC Mitochondrial Biology Unit, University of Cambridge, Cambridge CB2 0XY, UK; 4 Current affiliation: Department of Biochemistry and Molecular Biology B and Immunology, Biomedical Research Institute of Murcia (IMIB-Arrixaca), University of Murcia, Murcia 30120, Spain

## Abstract

Tumor necrosis factor (TNF) is a critical host resistance factor against tuberculosis. However, excess TNF produces susceptibility by increasing mitochondrial reactive oxygen species (mROS), which initiate a signaling cascade to cause pathogenic necrosis of mycobacterium-infected macrophages. Here, using the zebrafish, we identify the mechanism of TNF-induced mROS in tuberculosis. Excess TNF in mycobacterium-infected macrophages elevates mROS production by reverse electron transport (RET) through complex I. TNF-activated cellular glutamine uptake increases the Krebs cycle intermediate succinate. Oxidation of this elevated succinate by complex II drives RET, thereby generating the mROS superoxide at complex I. The complex I inhibitor, metformin, a widely used anti-diabetic drug, prevents TNF-induced mROS and necrosis of *Mycobacterium tuberculosis*-infected zebrafish and human macrophages, suggesting its utility in tuberculosis therapy.

## Abstract (summary)

**Introduction:**

Tumor necrosis factor (TNF) mediates resistance to tuberculosis. Excess TNF production, however, is detrimental because it induces pathogenic necrosis of infected macrophages in the tuberculous granuloma, which releases the mycobacteria into the extracellular milieu, promoting their growth and transmission to new hosts. Excess TNF, through the kinase RIP3 and the mitochondrial phosphatase PGAM5, increases reactive oxygen species such as superoxide and hydrogen peroxide in the mitochondria of mycobacterium-infected macrophages. These mitochondrial reactive oxygen species (mROS) initiate an elaborate interorganellar signaling circuit that ultimately causes macrophage necrosis and release of mycobacteria.

**Rationale:**

How TNF signaling elevates mROS production is not known. To address this question in vivo, we used zebrafish larvae, taking advantage of their optical transparency and their amenability to genetic and pharmacological manipulation. Thus, we could visualize and quantify mROS and macrophage necrosis after these manipulations.

**Results:**

Typically, mROS are generated during normal respiration when electrons from NADH, produced by metabolic pathways, enter the electron transport chain (ETC) and are transferred by forward electron transport from complex I to coenzyme Q (CoQ). We found that in wild-type animals (without excess TNF), mycobacterial infection induced a small boost in multiple metabolic pathways that increased mROS through this process. This slight increase in mROS did not result in macrophage necrosis. In animals with excess TNF (TNF^hi^ animals), we found that the greatly increased mROS were not induced through conventional forward electron transport but rather through reverse electron transport (RET). RET occurs when increases in the pool of reduced CoQ (CoQH_2_) from various metabolic pathways—in conjunction with a high proton motive force across the mitochondrial inner membrane—cause electrons to flow back through complex I instead of forward into complex III. RET can generate large amounts of mROS at complex I. We found that increased oxidation of succinate at complex II was responsible for RET mROS and that this metabolite was the source of the accumulation of CoQH_2_. Succinate is produced in the Krebs cycle, so we investigated its metabolic source. We found that TNF increased glutamine transport into the cell, boosting glutaminolysis, which increases the pool of α-ketoglutarate supplied to the Krebs cycle, resulting in increased succinate. Mycobacteria play a critical role in TNF-induced necrosis at two distinct steps. They were required together with TNF to increase glutaminolysis and then again with the resultant mROS to induce necrosis. By contrast, TNF had no further role in the necrosis pathway beyond inducing mROS. Thus, virulent mycobacteria have evolved multiple orchestrated mechanisms to exploit host genetic vulnerabilities (i.e., dysregulated TNF levels) to mediate macrophage necrosis as a way of increasing the transmission that is critical to their survival. Delineation of the TNF-induced RET mROS pathway identified several drugs already approved for other conditions that inhibit it at different steps. These drugs also inhibited TNF-induced macrophage necrosis and the animals’ hypersusceptibility to infection.

**Conclusion:**

RET, long thought to be an in vitro artifact, is now appreciated to play important homeostatic roles through moderate increases in mROS. However, excess RET has been shown to mediate the pathology associated with ischemia–reperfusion injury in heart attack and stroke. Our work shows that RET mROS also mediates tuberculosis pathology. Paradoxically, this means that the critical host determinant TNF can go from being protective to pathogenic depending on levels, context and the extent to which it can modulate host metabolism. Our prior work has shown that dysregulated TNF is pathogenic in human TB also. Therefore, the pathway-inhibiting drugs we have identified are promising host-targeting adjunctive drugs for tuberculosis, both drug-sensitive and drug-resistant. Metformin—a complex I inhibitor and widely used, well-tolerated anti-diabetic drug—is a particularly good candidate.

## Graphical Abstract

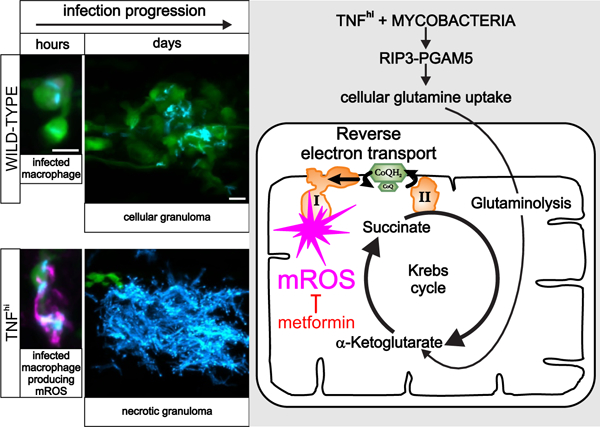

Excess TNF induces pathological mROS via RET in tuberculosis. **(Left)** TNF induces mROS (magenta) in macrophages (green) infected with mycobacteria (blue), which causes their necrosis with exuberant growth of released mycobacteria in the debris of the tuberculosis granuloma. (**Right**) Schematic depiction of how TNF works through RIP3 and PGAM5 to elevate mROS by RET through Complex I. Metformin inhibits Complex I to prevent TNF-induced mROS.

Tumor necrosis factor (TNF) is both a host resistance and susceptibility factor in tuberculosis (TB) (1–3). Findings in the genetically tractable and optically transparent zebrafish larva infected with *Mycobacterium marinum* (Mm) have revealed the mechanisms behind this dual effect (4–6). Although TNF is required for full microbicidal activity of mycobacterium-infected macrophages, its excess causes susceptibility by inducing their necrotic death, which releases mycobacteria into the growth-permissive extracellular environment (4, 7–9). This pathogenic role of dysregulated TNF was revealed through a zebrafish forward genetic screen, which found that both a deficiency and excess of leukotriene A4 hydrolase (LTA4H) cause susceptibility to Mm (4, 9). LTA4H catalyzes the synthesis of the pro-inflammatory leukotriene B4 (LTB_4_) and LTA4H/LTB_4_ deficiency and excess produce TNF deficiency and excess, respectively (4). These zebrafish studies led to the identification of a common, functional human *LTA4H* variant associated with mortality from tuberculous meningitis, the severest form of TB (4, 10). In cohorts in Vietnam and Indonesia, the high LTA4H-expressing variant was associated with increased cerebrospinal fluid TNF levels and increased mortality that was mitigated by adjunctive treatment with corticosteroids, broadly acting immunosuppressants (3, 4, 10). These findings implicated LTB_4_ and TNF-induced inflammation in mortality (3, 4, 10). Moreover, high TNF levels were associated with mortality even among individuals without the high LTA4H-expressing variant suggesting that TNF excess, resulting from diverse host genetic determinants, is a far-reaching host susceptibility factor in TB (3). Consistent with these findings, necrotic human tuberculous granulomas have more TNF than non-necrotic ones (11).

To gain mechanistic understanding of TNF-mediated pathogenic macrophage necrosis, we returned to the zebrafish larva. We found that excess TNF, acting through the kinase RIP3 and one of its substrates, PGAM5, increases mitochondrial reactive oxygen species (mROS) such as superoxide and hydrogen peroxide in infected macrophages (fig. S1) (5, 6). These mROS activate an interorganellar signaling circuit that involves the lysosome and the endoplasmic reticulum. This ultimately causes mitochondrial calcium overload, which then leads to necrosis (fig. S1) (5, 6). Here, by exploiting the zebrafish larva’s genetic and pharmacological tractability, we determine how TNF induces pathogenic mROS in mycobacterium-infected macrophages.

## TNF induces mROS through RET

**Figure 1:**
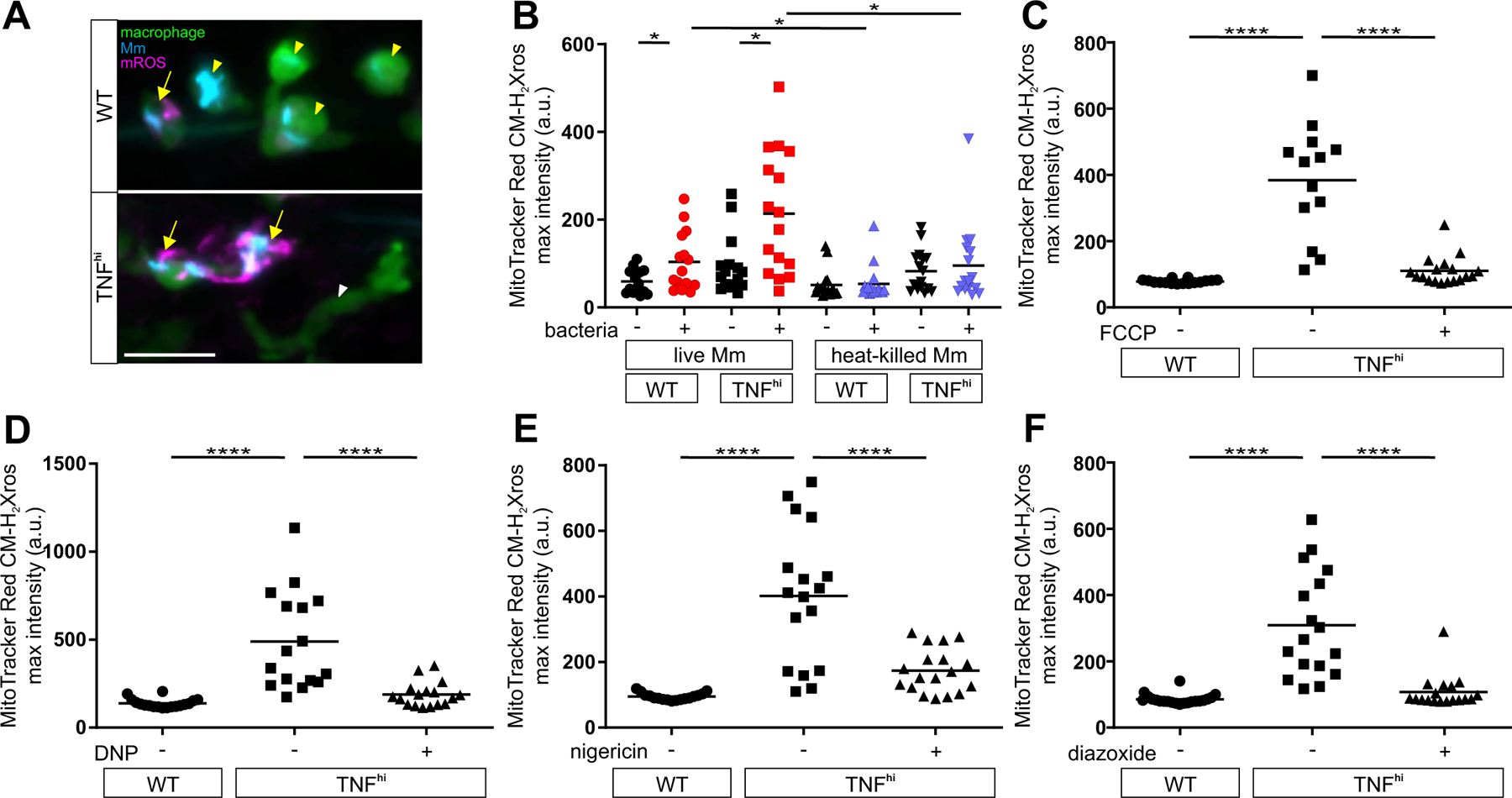
ETC-derived mROS drive necrosis of Mm-infected macrophages in TNF-high conditions. (A) Representative pseudocolored confocal images of wild-type (WT) or TNF^hi^ larvae with YFP-expressing macrophages (green), 1 day post infection (dpi) with EBFP2-expresssing Mm (blue), showing MitoTracker Red CM-H_2_Xros (magenta) fluorescence. White arrowheads, uninfected macrophages; yellow arrowheads, infected macrophages; yellow arrows, infected macrophages positive for mROS. Scale bar: 20 μm. (B) Quantification of mROS in wild-type or TNF^hi^ larvae 9 hours post-injection of live or heat-killed Mm. Each point represents the mean maximum intensity fluorescence of MitoTracker Red CM-H_2_Xros per fish. Black symbols represent macrophages that do not contain bacteria. Red and purple symbols represent Mm-infected and heat-killed Mm-containing macrophages, respectively, in the same animal. Horizontal bars, means; *P<0.05 (one-way ANOVA with uncorrected Dunn’s post-test for differences between macrophages in the same animal and with Tukey’s post-test for differences between treatments). Representative of two independent experiments. (C to F) Quantification of mROS in larvae 1 dpi with Mm that are wild-type, TNF^hi^ treated with (C) FCCP, (D) DNP, (E) nigericin, or (F) diazoxide, or vehicle. Horizontal bars represent means; ****P<0.0001 (one-way ANOVA with Tukey’s post-test). Representative of two-to-three independent experiments.

Administering exogenous TNF to Mm-infected zebrafish larvae phenocopies genetically induced TNF excess, causing macrophage necrosis and susceptibility by 5 days post-infection (fig. S2) (4). TNF selectively induces mROS in infected macrophages within 30 min, which rapidly trigger necrosis (5, 6). Using a general mitochondria-targeted ROS and oxidative stress sensor, we found that in wild-type animals, Mm infection alone causes 1.7–2.2-fold increases in mROS in infected macrophages compared to uninfected macrophages in the same animal (Fig. 1, A and B). In TNF^hi^ animals, mROS in infected macrophages were further increased to 3.6–6.6-fold over uninfected macrophages (Fig. 1, A and B). TNF did not increase mROS in uninfected macrophages, demonstrating that only infected macrophages were susceptible to TNF’s effects (Fig. 1, A and B). Moreover, heat-killed Mm failed to induce mROS in both wild-type and TNF^hi^ animals, suggesting that an actively synthesized (or heat-labile) bacterial determinant is needed (Fig. 1B). To confirm that the increased TNF^hi^ mROS originated from superoxide production by the electron transport chain (ETC), we asked if it was inhibited by compounds that disrupt mitochondrial electron transport. mROS were inhibited by four compounds that disrupt mitochondrial electron transport through distinct mechanisms (Fig. 1, C to F; fig. S2; and table S1). Thus, TNF-induced mROS originate in the ETC of mycobacterium-infected macrophages.

**Figure 2:**
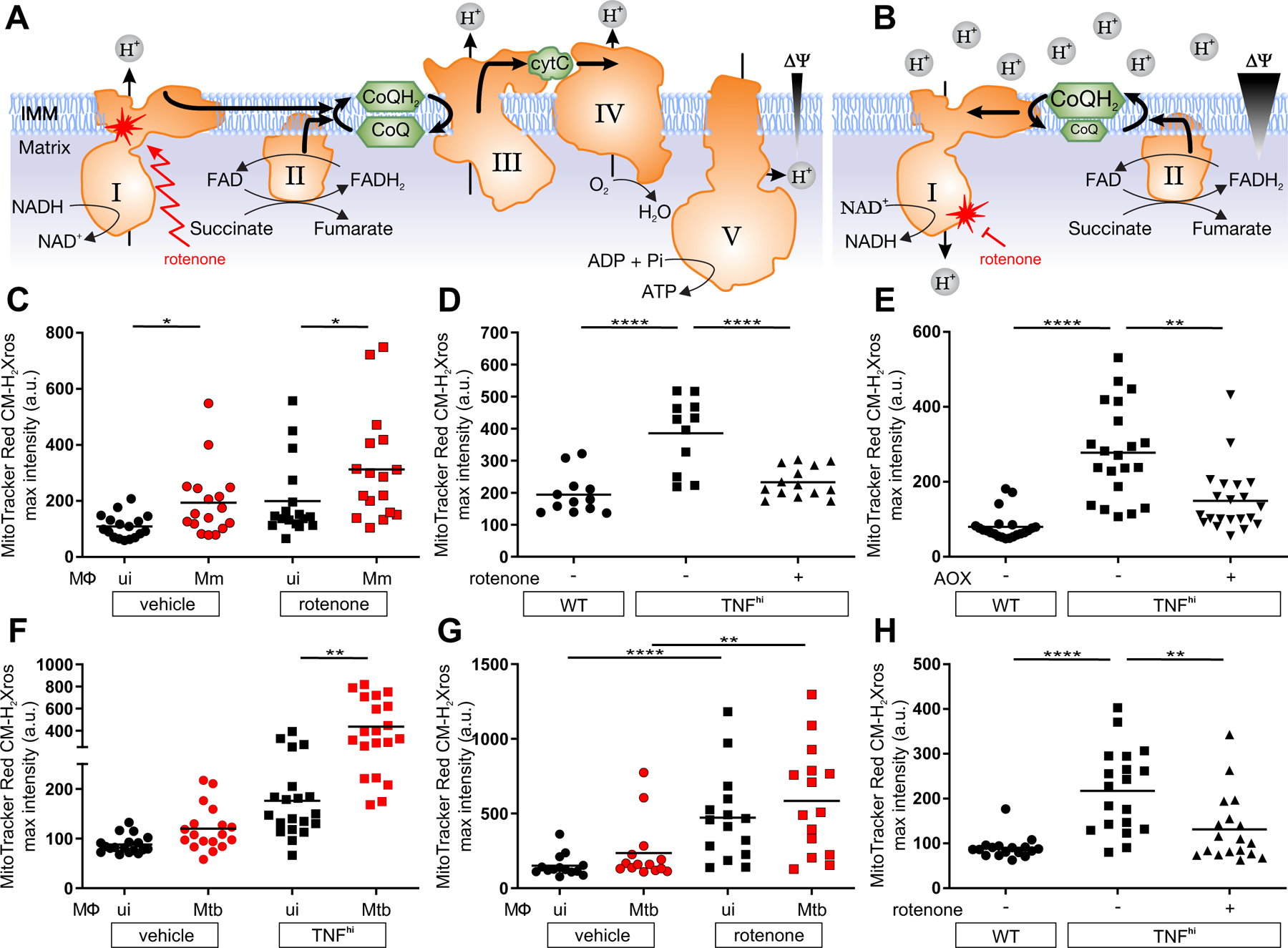
TNF induces RET mROS at complex I in mycobacterium-infected macrophages. (A and B) Illustrations of mROS production at complex I during (A) forward electron transport and (B) reverse electron transport. ΔΨ, membrane potential; IMM, inner mitochondrial membrane; I-V, complexes I-V; zigzag arrows, induction; red blunted arrows, inhibition. (C to H) Quantification of mROS in larvae 1 dpi with Mm (C to E) or Mtb (F to H) that are (C) wild-type treated with vehicle or rotenone, (D) wild-type (WT), TNF^hi^ treated with rotenone or vehicle, (E) wild-type, TNF^hi^, or TNF^hi^ expressing AOX, (F) wild-type or TNF^hi^, (G) wild-type treated with rotenone or vehicle, (H) wild-type, or TNF^hi^ treated with rotenone or vehicle. Horizontal bars represent means; *P<0.05, **P<0.01, ****P<0.0001 (one-way ANOVA with Dunn’s post-test (C, G, and H), Tukey’s post-test (D and E) or uncorrected Dunn’s post-test (F)). Black and red symbols in (C, F, and G) represent uninfected (ui) and infected macrophages, respectively, in the same animals. (C to G) representative of two-to-three independent experiments; (H) data from a single experiment.

During normal respiration, complex I receives electrons from NADH and transfers them to CoQ (Coenzyme Q), generating in the process a small amount of the mROS superoxide (O_2_^•−^) through single electron donation to O_2_ (12) (Fig. 2A). Increased O_2_^•−^ production at complex I is generated by two distinct mechanisms (12). In the first, disruption of electron transfer, due to ETC damage or loss of cytochrome C during apoptosis, results in an accumulation of NADH derived from multiple metabolic pathways. When electrons from NADH enter complex I and cannot flow forward towards ubiquinone, they generate O_2_^•−^ (Fig. 2A). In the second, increases in the extent of CoQ pool reduction (CoQH_2_) from various metabolic pathways, in conjunction with a high proton motive force across the mitochondrial inner membrane, cause electrons to flow back through complex I instead of forward into complex III (Fig. 2B) (13). This reverse electron transport (RET) by complex I generates mROS (O_2_^•−^ which dismutates to H_2_O_2_) (Fig. 2B). These two mechanisms can be distinguished by the effects of the complex I inhibitor rotenone, which increases mROS from forward electron flow through complex I but reduces mROS from RET (Fig. 2, A and B, and table S1) (12, 14). Rotenone increased mROS in the infected macrophages of wild-type animals, showing that they were generated by forward electron transport (Fig. 2C). By contrast, rotenone inhibited mROS in TNF^hi^ animals (Fig. 2D and table S1). Two other complex I inhibitors with different mechanisms of action also inhibited TNF^hi^ mROS (fig. S3 and table S1). Thus, TNF^hi^ mROS are generated by RET rather than by forward electron transport.

To corroborate that RET was responsible for TNF^hi^ mROS, we expressed *Ciona intestinalis* alternative oxidase (AOX) in TNF^hi^ larvae through injection of its mRNA. AOXs, which are present in plants, fungi, and some invertebrates but are absent in vertebrates, catalyze the transfer of electrons from the CoQH_2_ pool directly to O_2_, bypassing Complexes III and IV (fig. S4A) (15). AOX has been shown to prevent excessive reduction of the CoQ pool and mROS increases from RET (fig. S4A) (15). Thus, if TNF-induced mROS are generated by RET, they should be prevented by AOX expression (15). We confirmed that the *C. intestinalis* AOX was active in zebrafish by showing that AOX-expressing animals were resistant to cyanide, which poisons the ETC by inhibiting complex IV (fig. S4B) (15, 16). AOX expression decreased TNF^hi^ mROS, consistent with generation by RET from a reduced CoQ pool (Fig. 2, B and E).

Finally, we extended these findings to *Mycobacterium tuberculosis* (Mtb), the agent of human TB, using a leucine and pantothenic acid Mtb auxotroph that can be used in containment level 2 facilities (6). Mtb produced similar increases in mROS as Mm (Fig. 2F). Moreover, rotenone increased mROS in wild-type macrophages and inhibited TNF-induced mROS (Fig. 2, G and H). Thus, TNF-induced mROS increases in Mtb-infected macrophages are also derived from RET.

**Figure 3:**
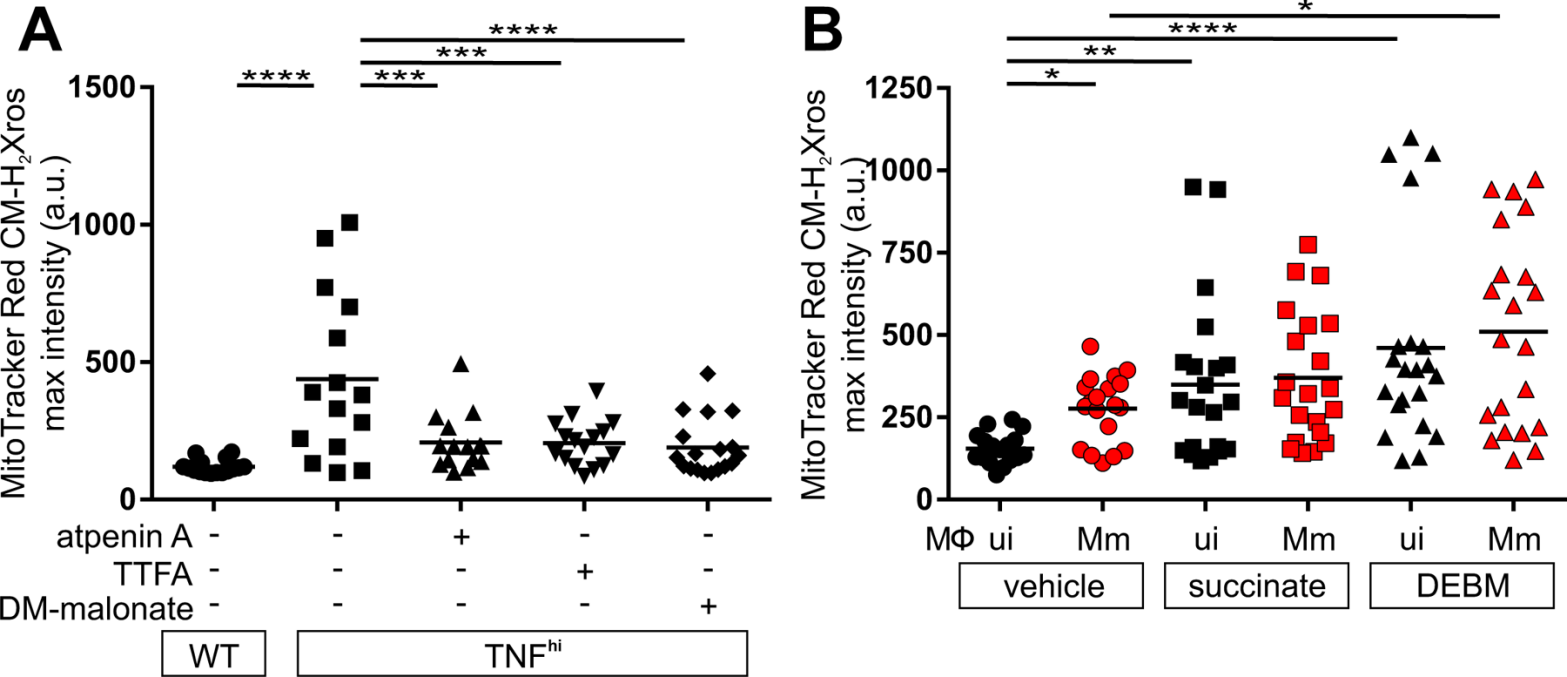
TNF increases succinate in mycobacterium-infected macrophages. Quantification of mROS in larvae 1 dpi with Mm that are (A) wild-type (WT), or TNF^hi^ treated with atpenin A, TTFA, DM-malonate, or vehicle (B) wild-type treated with succinate, DEBM, or vehicle. Horizontal bars represent means; *P<0.05; **P<0.01, ****P<0.0001 (one-way ANOVA with Tukey’s post-test (A) or Dunn’s post-test (B)). Black and red symbols in (B) represent uninfected (ui) and Mm-infected (Mm) macrophages, respectively, in the same animal. (A and B) representative of two-to-three independent experiments.

Although there are multiple sources of increased CoQH_2_, the most compelling candidate from both in vitro and in vivo studies was the increased oxidation of succinate at complex II (Fig. 2B) (13, 17). We tested this using three complex II inhibitors: atpenin A, TTFA, and dimethyl malonate (DMM), which is a prodrug of the competitive succinate dehydrogenase inhibitor malonate. All three inhibitors abolished mROS (Fig. 3A and table S1). If increased succinate oxidation at complex II was the source of RET and mROS, then increasing the mitochondrial succinate pool should have induced mROS even in wild-type animals in the absence of TNF^hi^ conditions. Diethyl succinate—a cell-permeable succinate ester known to increase mitochondrial succinate concentrations (18)—increased mROS in macrophages of wild-type animals (Fig. 3B). Diethyl butylmalonate (DEBM)—an inhibitor of the mitochondrial succinate transporter, which causes accumulation of endogenous mitochondrial succinate (19)—performed similarly (Fig. 3B). Thus, increased oxidation of succinate at complex II is necessary and sufficient for TNF-induced RET and mROS.

## TNF-activated glutaminolysis increases mitochondrial succinate

**Figure 4:**
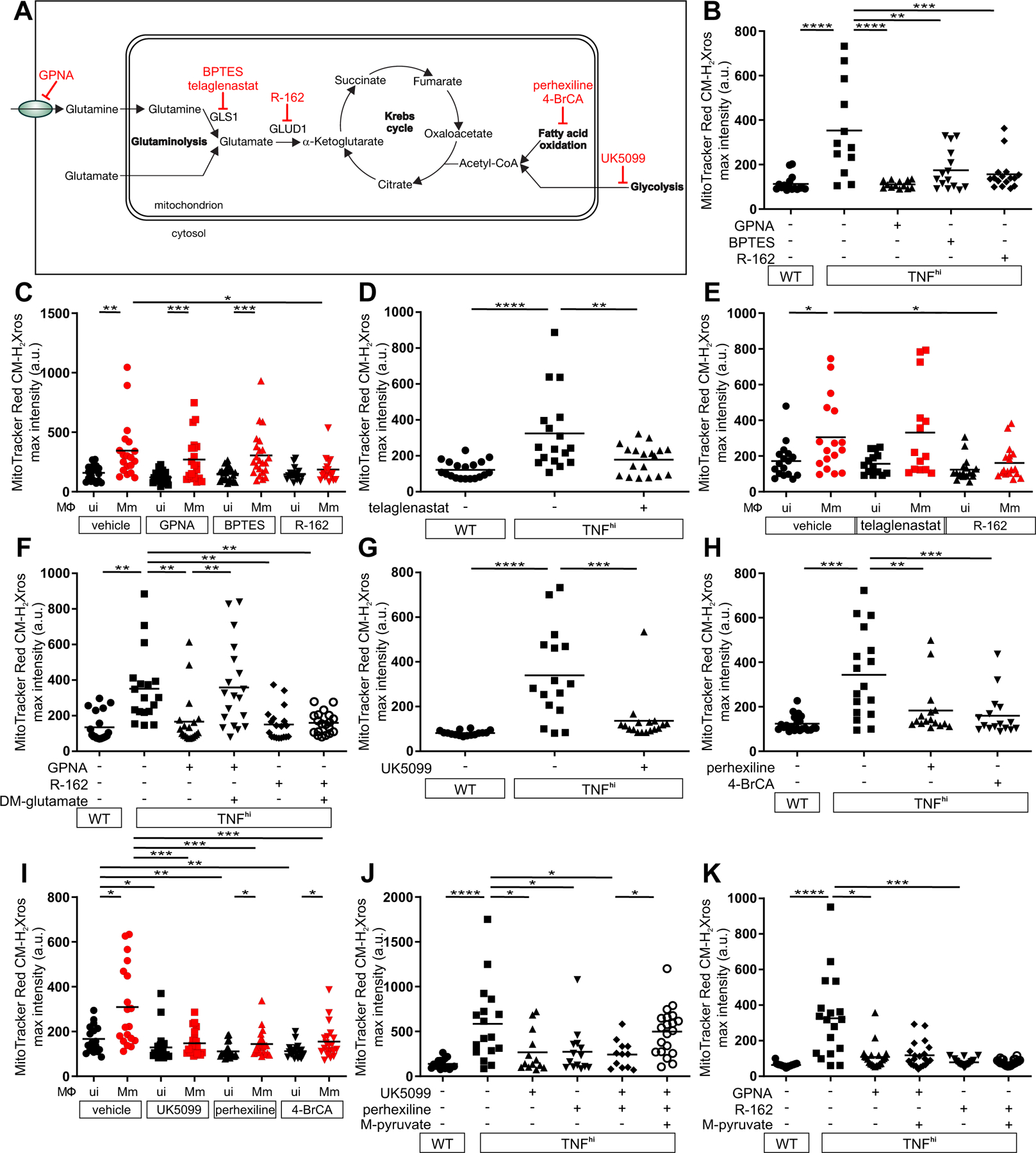
TNF-induced glutamine cellular uptake and increased glutaminolysis is responsible for RET and mROS production in mycobacterium-infected macrophages. (A) Illustration of main metabolic pathways fueling the Krebs cycle with inhibitors used (truncated red arrows). (B to K) Quantification of mROS in larvae 1 dpi with Mm that are (B) wild-type (WT) or TNF^hi^ treated with GPNA, BPTES, R-162, or vehicle, (C) wild-type treated with GPNA, BPTES, R-162, or vehicle, (D) wild-type or TNF^hi^ treated with telaglenastat or vehicle, (E) wild-type treated with telaglenastat, R-162, or vehicle, (F) wild-type or TNF^hi^ treated with vehicle, or GPNA or R-162 alone or in combination with DM-glutamate, (G) wild-type or TNF^hi^ treated with UK5099 or vehicle, (H) wild-type, or TNF^hi^ treated with perhexiline, 4-BrCA, or vehicle, (I) wild-type treated with UK5099, perhexiline, 4-BrCA, or vehicle, (J and K) wild-type or TNF^hi^ treated with vehicle, or UK5099 or perhexiline (J), or GPNA or R-162 (K) alone or in combination with M-pyruvate. Horizontal bars represent means; *P<0.05; **P<0.01, ***P<0.001, ****P<0.0001 (one-way ANOVA with Tukey’s post-test (B, D, F to H, J and K), Dunn’s post-test (C, E, and I)). Black and red symbols in (C, E, and I) represent uninfected (ui) and Mm-infected (Mm) macrophages, respectively, in the same animals. (B to D and G to I), representative of two-to-three independent experiments; (E, F, J, and K); data from a single experiment.

We next investigated the metabolic source of the increased succinate. Increased glycolysis, fatty acid oxidation, and glutaminolysis can all increase succinate by increasing Krebs cycle activity through increased input of pyruvate, acetyl-CoA, and α-ketoglutarate, respectively (Fig. 4A). We focused on glutaminolysis, which has been linked to TNF-mediated cell death (20, 21). Glutamine, the major amino acid transported in the circulation, is taken up into cells by multiple glutamine transporters and then into mitochondria where it is converted to glutamate and then α-ketoglutarate in the Krebs cycle (Fig. 4A). Four potential plasma membrane transporters that contribute to cellular glutamine uptake are highly expressed in human and zebrafish monocytes and macrophages (22–24). Of these, SLCA15 and SLC38A2 were identified in a screen for proteins phosphorylated by the RIP3 kinase in the context of necroptosis, a different form of TNF-mediated programmed cell death (25). Although distinct from necroptosis, our macrophage necrosis pathway also features RIP3, which is required for TNF-mediated mROS induction in mycobacterium-infected macrophages (fig. S1) (5, 6). Therefore, we tested GPNA, an inhibitor of both transporters (table S1). GPNA inhibited mROS in TNF^hi^ macrophages without affecting mROS in wild-type macrophages (Figure 4, A to C). We therefore hypothesized that TNF-RIP3-activated glutamine transport is the specific source of the increased mitochondrial glutamine for increased glutaminolysis, thereby increasing succinate. If correct, blocking the conversion of glutamine to glutamate should also specifically block TNF^hi^ mROS. Two different inhibitors of glutaminase 1 (GLS1), BPTES and CB-839 (telaglenastat), performed as expected, inhibiting mROS in TNF^hi^ but not wild-type macrophages (Fig. 4, A to E, and table S1). By contrast, when the conversion of glutamate to α-ketoglutarate was inhibited using R-162, mROS was inhibited in both TNF^hi^ and wild-type macrophages (Fig 4, A to C, E and table S1). Thus, although the smaller increase in mROS from infection alone also requires glutaminolysis, it can be sustained by mitochondrial glutamate transported directly from the cytosol where it is produced through transamination reactions (Fig. 4, C and E). Finally, to confirm the specificity of GPNA and R-162 in our system, we used each inhibitor in combination with dimethyl glutamate, a cell permeable source of glutamate (table S1). Dimethyl glutamate restored GPNA-inhibited mROS but not R-162-inhibited mROS (Fig. 4F). Thus, TNF stimulation of infected macrophages specifically activates glutamine uptake to increase glutaminolysis to induce mROS.

If high TNF also increases glycolysis and/or fatty acid oxidation (Fig. 4A), then inhibiting these pathways should also specifically inhibit TNF^hi^ but not wild-type mROS. However, inhibition of mitochondrial pyruvate transport using UK5099, or fatty acid oxidation using perhexiline or 4-bromocrotonic acid (4-BrCA) removed mROS in both wild-type and TNF^hi^ animals (Fig. 4, G to I, and table S1). We confirmed the specificity of UK5099 and perhexiline by showing that methyl pyruvate, a cell-permeable pyruvate derivative restored mROS inhibited by them but not by GPNA or R-162 (Fig. 4, J and K). Thus, TNF and infection together activate cellular glutamine uptake and the resultant increase in glutaminolysis is the specific source of the increased succinate. Because oxidation of excess succinate would increase the levels of the downstream intermediates malate and oxaloacetate (a potent complex II inhibitor) (26), glycolysis and fatty acid oxidation would be required to play a “supporting role” by providing acetyl-CoA to consume oxaloacetate. Thus, the build-up of oxaloacetate would be prevented, allowing continued complex II activity (Fig. 4A).

**Figure 5:**
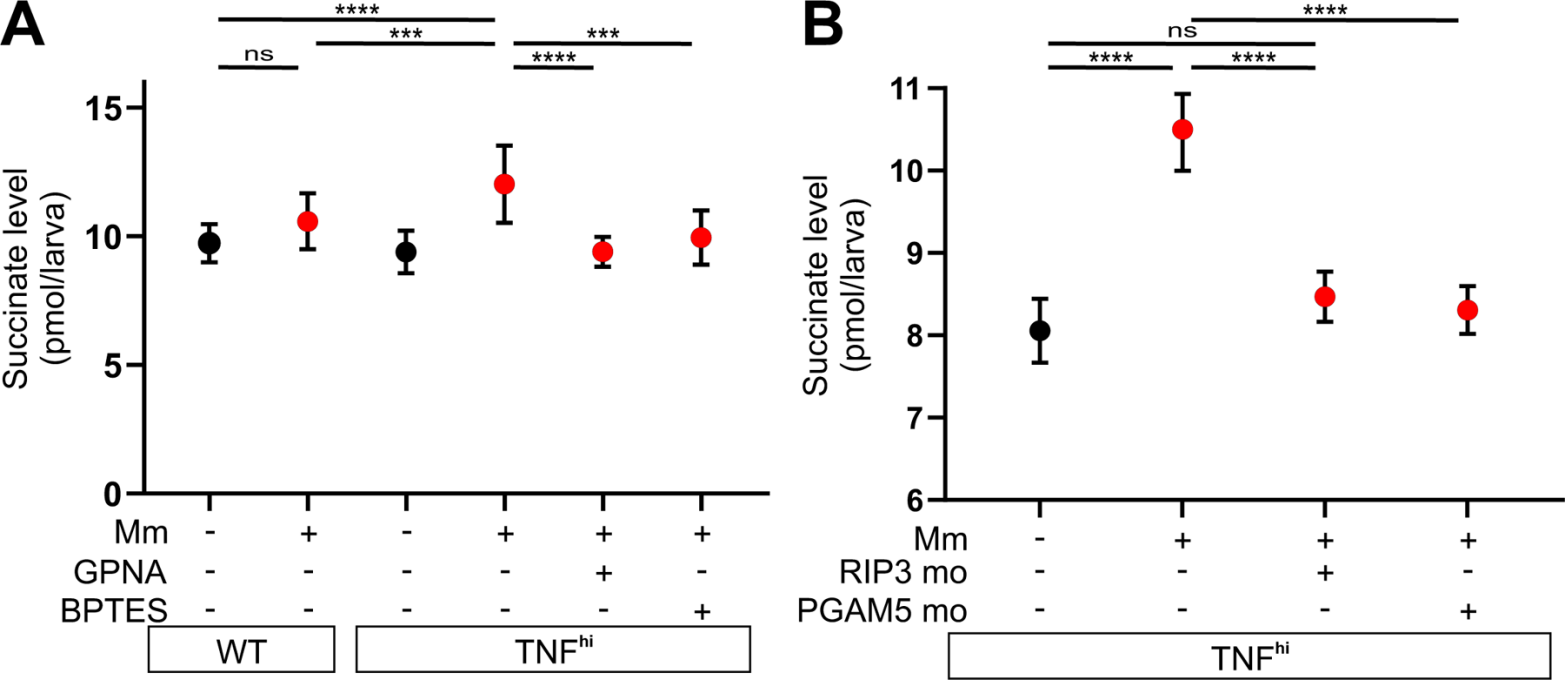
TNF-induced glutaminolysis increases succinate levels in mycobacterium-infected macrophages in a RIP3- and PGAM5-dependent manner. (A and B) Quantification of succinate in zebrafish larvae 1 dpi with Mm or mock-injected, that are (A) wild-type (WT) or TNF^hi^ treated with GPNA, BPTES, or vehicle and (B) TNF^hi^, TNF^hi^ RIP3 morphants, or TNF^hi^ PGAM5 morphants. Each point represents the mean of four independent experiments in A and two independent experiments in B. Horizontal bars represent pooled SD. ***P<0.001, ****P<0.0001 (one-way ANOVA with Tukey’s post-test).

We used liquid chromatography–mass spectrometry to quantify succinate levels in the larvae under the different conditions. Infection and TNF combined (but neither alone) increased succinate levels over baseline (Fig. 5A and data S1). Moreover, GPNA and BPTES inhibited this increase, as predicted (Fig. 5A and data S1). Although further validation of the source of succinate by measurement of flux to it from stable isotope labeled precursors such as glutamine was not technically possible in this in vivo system, our findings that both mROS and succinate levels increase in the TNF^hi^ state and decrease to wild-type levels upon inhibiting glutamine uptake or its conversion to glutamate provide strong evidence that glutaminolysis from increased glutamine transport is the source of the increased succinate. As with mROS increases, these succinate increases also occurred rapidly within 30 min of TNF administration. The rapid induction of succinate and mROS is consistent with TNF–RIP3-induced post-translational modifications (e.g., phosphorylation), as previously proposed (20, 21). Accordingly, RIP3 knockdown inhibited TNF-induced succinate in infected animals (Fig. 5B and data S1). Finally, TNF-induced succinate was also inhibited by knockdown of PGAM5, a mitochondrial phosphatase, which is required together with RIP3 both for TNF-mediated necroptosis (27) and for TNF-induced mROS and necrosis of mycobacterium-infected macrophages in our pathway (Fig. 5B, data S1 and fig. S1) (5, 6). Thus, TNF signals via RIP3 and PGAM5 to activate glutamine transport to increase glutaminolysis and Krebs cycle succinate.

## TNF, mROS, and mycobacteria play discrete roles in macrophage necrosis

We dissected the interactions between TNF, mROS, and mycobacteria and what roles they play at distinct steps of the pathway. We had shown that both TNF and mycobacteria are required to increase mitochondrial succinate, which is required to induce mROS. Because exogenous succinate could induce mROS in wild-type animals in both infected and uninfected macrophages (Fig. 3B), we concluded that the only role for TNF and mycobacteria in mROS induction in this system is to increase mitochondrial succinate.

**Figure 6:**
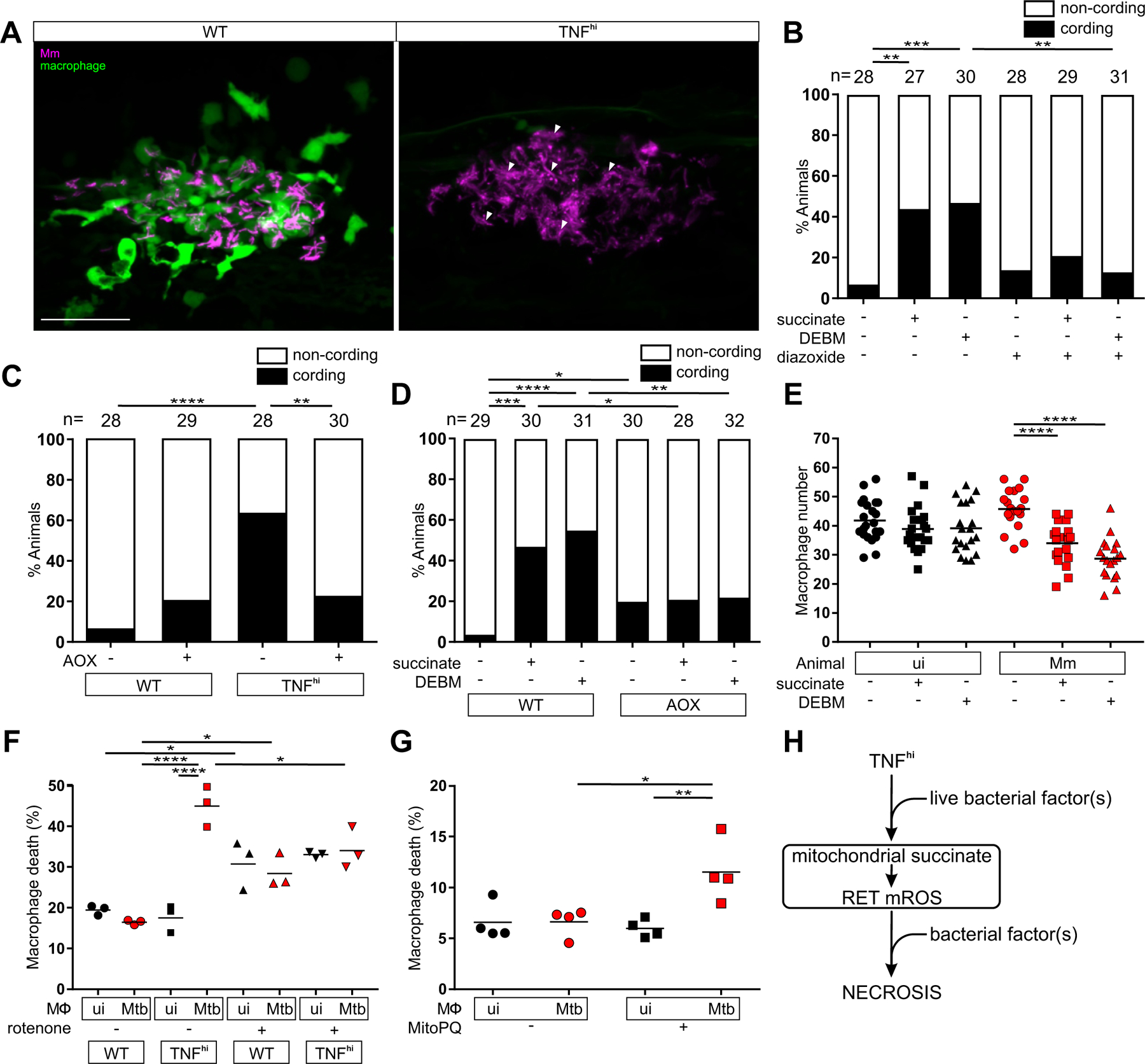
TNF-mediated increased glutamine cellular uptake in mycobacterium-infected increases succinate oxidation, mROS and necrosis. (A) Representative pseudocolored confocal images of 5 dpi granulomas in wild-type (WT) or TNF^hi^ larvae with YFP-expressing macrophages (green) infected with tdTomato-expressing Mm (magenta). Arrowheads, extracellular cording bacteria. Scale bar: 50 μm. (B) Bacterial cording in wild-type larvae 5 dpi with Mm, treated with vehicle, or succinate or DEBM alone or in combination with diazoxide; **P<0.01, ***P<0.001 (Fisher’s exact test). (C) Bacterial cording 5 dpi with Mm in wild-type and TNF^hi^ larvae and wild-type and TNF^hi^ larvae expressing AOX; **P<0.01, ****P<0.0001 (Fisher’s exact test). (D) Bacterial cording 5 dpi wild-type or AOX-expressing larvae infected with Mm and treated with succinate, DEBM, or vehicle; *P<0.05; **P<0.01, ***P<0.001, ****P<0.0001 (Fisher’s exact test). (E) Number of trunk macrophages in Mm-infected (Mm) larvae and mock-injected (ui) larvae 1 dpi. Horizontal bars represent means; ****P<0.0001 (one-way ANOVA with Dunn’s post-test). (F and G) Percentage of dead THP-1 macrophages at 5 hours post-TNF, treated with (F) rotenone or vehicle starting 1 hour before TNF addition or (G) MitoParaquat (MitoPQ) or vehicle for 5 hours. Black and red symbols represent uninfected (ui) and Mtb-infected macrophages (Mtb), respectively, within the same treatment well. Horizontal bars represent means; *P<0.05, **P<0.01, ****P<0.0001 (one-way ANOVA with Tukey’s post-test). (H) Schematic diagram showing the role of TNF, mROS and mycobacterial factor(s) in TNF-mediated necrosis of mycobacterium-infected macrophages. (C, D to E, and G) representative of two independent experiments; (B and F) data from a single experiment.

We have previously shown that the mROS are required for macrophage necrosis (5). We now asked whether they were sufficient to complete macrophage necrosis or whether TNF and/or mycobacteria further required downstream of mROS induction. Macrophage necrosis results in exuberant extracellular mycobacterial growth in characteristic cords (Fig. 6A). Bacterial cording can be used as a reliable surrogate marker for infected macrophage death (9). We found that both exogenous succinate and DEBM induced the necrosis of infected macrophages as evidenced by increased bacterial cording (Fig. 6B). This necrosis was a direct consequence of RET mROS production, as disrupting the ETC with diazoxide reduced cording (Fig. 6B). Moreover, bypassing the ETC by AOX expression—which decreased TNF-induced mROS (Fig 2E)—inhibited both TNF-mediated macrophage necrosis (Fig 6C) as well as succinate- and DEBM-induced necrosis in wild-type animals (Fig. 6D). Thus, TNF plays no further role in macrophage necrosis beyond increasing mitochondrial succinate.

To determine if mycobacteria were required for necrosis downstream of mROS induction, we examined if diethyl succinate and DEBM could also kill uninfected macrophages by enumerating macrophages in infected and uninfected animals (5). Diethyl succinate and DEBM reduced macrophage numbers only in the infected animals, suggesting that, in contrast to TNF, mycobacteria are required downstream of mROS to induce necrosis (Fig. 6E).

Similar results were observed in human macrophages derived from the monocytic cell line THP-1. We had previously shown that TNF induces necrosis in Mtb-infected THP-1 cells through the same interorganellar pathway downstream of mROS as in Mm-infected zebrafish (fig. S2) (6). We used rotenone to confirm that RET was responsible for mROS induction in these cells. In the absence of TNF, rotenone increased death of both infected and uninfected cells, as expected from the oxidative stress it induces, but there was a specific reduction of TNF-induced death of infected macrophages (Fig. 6F). Next, to test our findings from zebrafish concerning about the role of TNF, mROS, and mycobacteria, we treated Mtb-infected THP-1 cells with MitoParaquat (MitoPQ), a mitochondria-targeted compound that produces superoxide through redox cycling at the complex I flavin site (table S1). MitoPQ increased necrosis in the absence of TNF but only in infected macrophages (Fig. 6G and fig. S2). This confirmed that TNF has no further role in the necrosis pathway beyond inducing mROS whereas mycobacteria are required downstream of mROS induction. By contrast, one or more mycobacterial factors shared between Mm and Mtb operate at two distinct points in this pathway: first to enable TNF-mediated mROS by activating cellular glutamine uptake and increasing mitochondrial succinate to produce complex II-mediated RET-ROS and then to promote the necrosis of macrophages experiencing this mROS (Fig. 6H).

## mROS pathway reveals host-targeting drugs for TB

**Figure 7:**
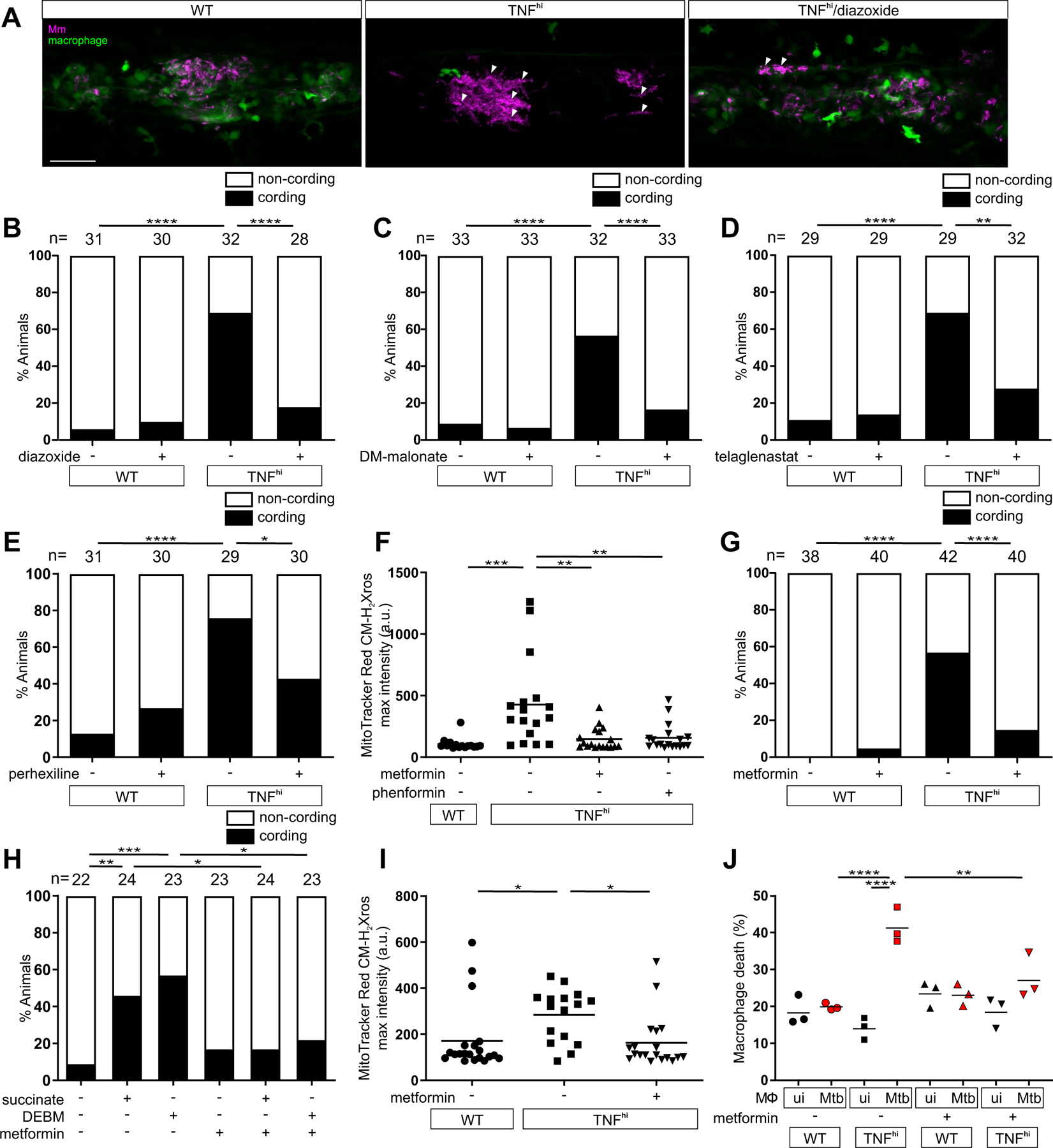
Currently available drugs can intercept TNF-induced mROS production and inhibit necrosis of mycobacterium-infected macrophages. Representative pseudocolored confocal images of 5 dpi granulomas in larvae with yellow fluorescent macrophages (green) that are wild-type (WT), or TNF^hi^ treated with diazoxide or vehicle, infected with red fluorescent Mm (magenta). Arrowheads, extracellular cording bacteria. Scale bar: 50 μm. (B to E) Bacterial cording in wild-type or TNF^hi^ larvae 5 dpi with Mm, treated with vehicle or (B) diazoxide, (C) DM-malonate, (D) telaglenastat, or (E) perhexiline. *P<0.05; **P<0.01, ****P<0.0001 (Fisher’s exact test). (F) Quantification of mROS in wild-type or TNF^hi^ larvae 1dpi with Mm, treated with metformin, phenformin, or vehicle. Horizontal bars represent means; **P<0.01; ***P<0.001 (one-way ANOVA with Tukey’s post-test). (G) Bacterial cording in wild-type or TNF^hi^ larvae 5 dpi with Mm, treated with metformin or vehicle. ****P<0.0001 (Fisher’s exact test). (H) Bacterial cording in wild-type larvae 5 dpi with Mm, treated with vehicle, or succinate or DEBM alone or in combination with metformin. *P<0.05; **P<0.01, ***P<0.001 (Fisher’s exact test). (I) Quantification of mROS in wild-type or TNF^hi^ 1 dpi with Mtb, treated with metformin or vehicle. Horizontal bars represent means; *P<0.05 (one-way ANOVA with Tukey’s post-test). (J) Percentage of dead THP-1 macrophages at 5 hours post-TNF, treated with metformin or vehicle starting 1 hour before TNF addition. Black and red symbols represent uninfected (ui) and Mtb-infected macrophages (Mtb), respectively, within the same treatment well. Horizontal bars represent means; **P<0.01, ****P<0.0001 (one-way ANOVA with Tukey’s post-test). (B to G, and I) representative of two independent experiments; (H and J) data from a single experiment.

We had previously shown that blocking mROS using scavengers such as N-acetyl cysteine inhibited TNF-induced macrophage necrosis and restored resistance (5). Four of the compounds used here to inhibit mROS, and thus delineate the mechanism of mROS production, are approved oral drugs or under investigation for other conditions. We therefore assessed if these drugs also inhibited macrophage necrosis (fig. S5). These included diazoxide, a disruptor of electron transport that is approved for hyperinsulinemic hypoglycemia; perhexiline, a mitochondrial carnitine palmitoyltransferase-1 inhibitor that is approved for angina; telaglenastat, a GLS1 inhibitor that is in clinical trials for cancer; and DMM, the complex II inhibitor that has been shown to prevent ischemia-reperfusion injury in models of heart attack (fig. S5 and table S1) (28). All four inhibited TNF-mediated macrophage necrosis in the zebrafish (Figure 7, A to E). We then asked if metformin, a widely-used antidiabetic drug that inhibits Complex I (fig. S5 and table S1) (29), could be a potential host-targeting drug to prevent TNF-induced pathogenic macrophage necrosis in TB. Metformin inhibited TNF-elicited mROS in Mm-infected larvae as did its more hydrophobic derivative phenformin (Fig. 7F). Metformin also inhibited TNF-mediated necrosis of Mm-infected macrophages (Fig. 7G). Moreover, it also inhibited Mm-infected macrophage necrosis resulting from increased mitochondrial succinate (Fig. 7H). Thus, although metformin has pleiotropic effects and is a relatively weak complex I inhibitor (29), it specifically inhibits TNF-mediated necrosis by blocking RET-generated mROS at Complex I. Finally, metformin inhibited mROS in the infected macrophages of Mtb-infected zebrafish (Fig. 7I) and inhibited necrosis of Mtb-infected THP-1 cells (Fig. 7J), confirming that its inhibitory activity was relevant in the context of Mtb infection.

## DISCUSSION

Though long thought to be an in vitro artifact, moderate levels of RET and resultant increases in mROS have important homeostatic roles in cell differentiation and oxygen sensing (13). However, excess RET has pathological roles in ischemia–reperfusion injury of the heart and brain (18, 30). During ischemia, rewiring of the Krebs cycle reduces fumarate levels, leading to succinate accumulation (18). During the reperfusion phase, rapid oxidation of the accumulated succinate triggers RET and mROS, which causes tissue necrosis leading to irreparable organ damage (18, 30). The TNF-mediated necrosis pathway described here has two significant differences. First, the source of the succinate is different and second, in ischemia–reperfusion injury, the mROS alone appear sufficient to drive necrosis whereas a second “hit” in the form of one or more bacterial determinants is required in our TNF-induced macrophage necrosis pathway. Perhaps the inflammatory milieu generated during ischemia generates the additional signal(s) that combine with mROS to cause necrosis.

We also considered our findings in the light of work using cultured macrophages, which has shown that succinate is responsible for generating proinflammatory responses to lipopolysaccharide (LPS), a key virulence determinant of Gram-negative bacteria (19). LPS causes macrophages to switch to aerobic glycolysis while generating succinate from enhanced glutaminolysis by an undescribed means. Succinate induces mROS, likely through RET, and these mROS drive pro-inflammatory cytokines via HIF1α stabilization (19, 31). This sequence contrasts with the pathway described here where TNF is upstream, not downstream, of mROS and TNF is not among the cytokines induced by LPS and succinate. Thus, distinct pathogenic determinants specific to Gram-negative bacteria and mycobacteria—a cell wall constituent versus a product of live mycobacteria—channel mROS to produce discrete cellular responses.

We were particularly interested in pursuing this TNF-mediated necrosis pathway because of its clinical implications. Currently, tuberculous meningitis is treated with adjunctive corticosteroids which are broadly immunosuppressive and have multiple additional serious adverse effects. Our prior studies on the TNF-mediated necrosis pathway identified several pathway-specific drugs that inhibit macrophage necrosis without being broadly anti-inflammatory, all with a decades-long history of use in humans for other conditions (5, 6). This work now identifies additional drugs, including the widely used oral antidiabetic drug, metformin. Metformin readily crosses the blood-brain barrier, resulting in high brain and CSF concentrations (table S1) (32). This highlights its potential therapeutic utility in tuberculous meningitis. Metformin was reported to ameliorate Mtb infection in mice via diverse mechanisms, including broadly acting anti-inflammatory effects and to enhance the efficacy of antitubercular antibiotics in one but not another study, leading to an ongoing trial as an adjunctive agent for lung TB (33–36). Adjunctive corticosteroid treatment has been suggested to reduce inflammation and bacterial burdens in lung TB, the most common, contagious form that sustains the global disease burden (37, 38). It will be interesting to see whether metformin particularly benefits individuals with the high *LTA4H* genotype, and, given the association of TNF with necrotic lung granulomas (11), whether it has a particular benefit in resolving necrotic lesions.

## MATERIALS AND METHODS

### Zebrafish husbandry and infections

Zebrafish husbandry and experiments were conducted in compliance with guidelines from the UK Home Office using protocols approved by the Animal Welfare and Ethical Review Body of the University of Cambridge. Zebrafish AB wild-type strain (Zebrafish International Resource Center) (ZFIN ID: ZDB-GENO-960809–7) and the transgenic line *Tg(mpeg1:YFP)*^*w200*^ (with yellow fluorescent macrophages) (ZFIN ID: ZDB-FISH-150901–6828) (6) in the AB background were used. All zebrafish lines were maintained in buffered reverse osmotic water systems as previously described (6). Zebrafish embryos were housed at 28.5°C in fish water from collection to 1 day post-fertilization (dpf) and in E2 Embryo Medium diluted to 0.5X (E2/2) supplemented with 0.003% 1-phenyl-2-thiourea (PTU) (Sigma) from 1 dpf to prevent pigmentation (6). Larvae (of undetermined sex given the early developmental stages used) were anesthetized, infected at 2 dpf via caudal vein (CV) injection for all assays, and randomly allotted to the different experimental conditions as previously described (6, 59). Sample size was determined based on previous similar experiments or on pilot experiments.

### Bacterial strains

Mm M strain (ATCC #BAA-535) and Mtb H37Rv strain, mc^2^6206 *ΔleuD ΔpanCD* (60) expressing tdTomato, mWasabi, or EBFP2 were grown as previously described (59, 61). For experiments to assay bacterial cording and number of macrophages in the trunk of the animal, zebrafish larvae were infected with 150–200 tdTomato-expressing Mm. To assess mROS, larvae were infected with 90–120 EBFP2-expressing or 84 mWasabi-expressing Mm, 80–100 EBFP2-expressing Mtb, or injected with 336 heat-killed mWasabi-expressing Mm (heat-killed by incubation at 80°C for 20 min). To assess succinate levels, zebrafish larvae were infected with 200–300 tdTomato-expressing Mm.

### TNF and drug administration to zebrafish larvae

TNF^hi^ animals were created by injecting recombinant zebrafish soluble TNF (62) as previously described (4). To assess drug treatment in infected fish, equivalently infected sibling larvae were mixed in a Petri dish and held at 28.5°C before random allocation to the drug-treated or control groups; 0.5% DMSO (Sigma) was used as the control (vehicle). Drugs dissolved in DMSO or water were kept in small aliquots at −20°C before administration to 1 dpi larvae by adding them to the water (E2/2 medium). Doses used in this work were based on previous studies or pilot experiments, using the minimum effective concentration without deleterious or toxic effects on larvae for the duration of the experiment (see table S2). FCCP (carbonyl cyanide-4-(trifluoromethoxy)phenylhydrazone) (50 nM) (Cambridge Bioscience) was administered 1.5 hours before MitoTracker Red CM-H2-Xros injection. TTFA (thenoyltrifluoroacetone) (1 μM) (Cambridge Bioscience), atpenin A5 (2.5 nM) (Insight Biotechnology), diethyl succinate (500 nM) (reagent plus 99% Sigma), and DEBM (diethyl butyl malonate) (1 μM) (Sigma) were administered 2 hours before MitoTracker Red CM-H2-Xros injection. DM-Glutamate (dimethyl glutamate) (60 μM) (Cambridge Bioscience) was administered 3 hours before MitoTracker Red CM-H_2_-Xros injection. DNP (2.4-dinitrophenol) (100 nM) (Agilent Technologies) was administered 3.5 hours before MitoTracker Red CM-H_2_-Xros injection. Rotenone (6.25 nM) (Sigma), piericidin A (50 nM) (Stratech Scientific), strobilurin B (100nM) (Insight Biotechnology), metformin (20 μM) (VWR International), phenformin (20 μM) (Sigma), nigericin (5 μM) (Sigma), diazoxide (50 nM) (Cambridge Bioscience), UK5099 (10 μM) (Cambridge Bioscience), and M-pyruvate (methyl pyruvate) (50 nM) (Fisher Scientific) were administered 4 hours before MitoTracker Red CM-H_2_-Xros injection. DM-malonate (dimethyl malonate) (10 μM) (Sigma), perhexiline (10 μM) (Stratech Scientific), 4-BrCA (4-bromocrotonic acid) (10 μM) (Insight Biotechnology), GPNA (10 μM) (Cambridge Bioscience), BPTES (5 μM) (Cambridge Bioscience), telaglenastat (5 μM) (Cambridge Bioscience), and R-162 (1 μM) (Cambridge Bioscience) were administered 5 hours before MitoTracker Red CM-H_2_-Xros injection. In experiments to assess cording, perhexiline was removed 5 hours after TNF administration, diethyl succinate and DEBM were administered for 10 hours and then removed, and metformin, phenformin, DM-malonate, diazoxide, and telaglenastat were added 1 dpi and removed 2 dpi. After drug removal, the larvae were maintained in fresh E2/2 medium for the rest of the experiment. In experiments to assess macrophage numbers, diethyl succinate and DEBM were administered 1 dpi for 24 hours until macrophage number was assessed 2 dpi. For experiments quantifying mitochondrial ROS production, drugs were added before MitoTracker Red CM-H_2_-Xros injection as indicated above and maintained during imaging.

### Synthetic mRNA synthesis and microinjection

The ORF sequence of the alternative oxidase (AOX) from *Ciona intestinalis* was obtained by PCR using as a template the plasmid MAC_C_AOX (Addgene plasmid# 111661). The T7 promoter (5′-TAATACGACTCACTATAGG-3′) followed by the zebrafish Kozak sequence 5′-GCCGCCACC-3′ were inserted before the start codon by PCR. mRNA was synthesized using the mMessage mMachine kit (Ambion) and the polyA Tailing kit (Ambion). Approximately 2–4 nl of injection solution (4) containing 200 μg/ml of AOX mRNA was injected into the yolks of embryos at the one-to-two-cell stage.

### Morpholino-mediated knockdown of RIP3 and PGAM5

RIP3 e2/i2-splice-blocking (5′-TTTTAGAAATCACCTTGGCATCCAG-3′) and PGAM5-translation-blocking morpholino (5′-AGCGCCCTCCGAAAAGACATGCTTC-3′) (Gene Tools) were diluted to 0.15 mM in injection solution (4). Approximately 2–4 nl was injected into the yolks of embryos at the one-to-two-cell stage.

### Heart rate assessment of zebrafish larvae

AOX-expressing 2 dpf larvae were treated with different concentrations of KCN for an hour. Heart rate (beats per minute) was assessed as a readout of cyanide poisoning of complex IV of the electron transport chain (63) in absence of anesthetic using a dissecting microscope.

### Zebrafish larvae microscopy

Fluorescence microscopy was performed as described (59). Mycobacterial cording and macrophage numbers were assessed in the trunk of the larvae using a Nikon Eclipse E600 upright microscope fitted with Nikon Plan Fluor 10X 0.3 NA and Nikon Plan Fluor 20X 0.5 NA objectives. For laser scanning confocal microscopy, anesthetized larvae were embedded in low-melting-point agarose as previously described (6). A Nikon A1R confocal microscope with a Plan Apo 20X 0.75 NA objective was used to generate 35–40 mm *z*-stacks consisting of 0.3–2-mm optical sections. The galvano scanner was used for all static imaging and for time-lapse imaging of the caudal hematopoietic tissue (CHT, area located between the cloaca and the beginning of the caudal fin). Images were acquired with NIS Elements (Nikon). A heating chamber (Oko-labs) adapted to the microscope was used to maintain temperature at 28.5°C during imaging. Confocal images are pseudocolored to facilitate visualization.

### Mitochondrial ROS quantification assay in zebrafish larvae

Mitochondrial ROS production was assayed by fluorescence intensity of MitoTracker Red CM-H_2_-Xros, a cell-permeable fluorogenic probe for ROS which is targeted to the mitochondrion and produces red fluorescence upon oxidation by diverse ROS (Fisher Scientific) (5, 6). *Tg(mpeg1:YFP)*^*w200*^ larvae were infected 2 dpf. For all experiments where TNF^hi^ animals are used, larvae were microinjected 1 dpi via CV with phosphate buffered saline (PBS) containing TNF and 50 mM MitoTracker Red CM-H_2_-Xros or PBS containing vehicle for TNF and MitoTracker Red CM-_H2_-Xros (6). For experiments where mROS production was quantified in mycobacterium-infected versus uninfected macrophages, 100 mM MitoTracker Red CM-H_2_-Xros was used instead to increase sensitivity of the probe. After administration of MitoTracker Red CM-H_2_-Xros (in combination with TNF or alone), larvae were prepared for confocal imaging and maintained at 28.5°C within a heated incubation chamber attached to the confocal microscope. Images of the CHT of each larva were taken starting 30–60 min after MitoTracker Red CM-H_2_-Xros administration. Mitochondrial ROS production was quantified using maximum projection images as MitoTracker Red CM-H_2_-Xros maximum fluorescence intensity per macrophage using NIS-Elements. When not otherwise stated in the figure legend, the mean of maximum MitoTracker Red CM-H_2_-Xros fluorescence was quantified only in Mm- or Mtb-infected macrophages.

### Succinate quantification by liquid chromatography-mass spectrometry

Three to six pools of 20 1-dpi larvae per condition per experiment were collected and flash frozen 30 min after TNF injection, with the time set after injecting 75% of the larvae for each experimental group. Each pool was homogenized in 300 µl of extraction buffer and succinate was quantified as described (64). The means and pooled standard deviations of independent experiments were calculated and compared using one-way ANOVA with Tukey’s post-hoc multiple comparisons test.

### Quantification of THP-1 cell necrosis

THP-1 cells (ATCC TIB-202) were differentiated into macrophages and infected with single-cell suspensions of mCherry- or tdTomato-expressing Mtb mc^2^6206 *ΔleuD ΔpanCD* as described (6). In THP-1 experiments with added TNF, 1-day post-infection cells were pre-incubated with 10 nM rotenone, 1 mM metformin or 0.1% DMSO vehicle control for 1 hour. Human recombinant TNF (Sigma) in a solution of 5% trehalose/PBS (Sigma) was then added to treatment wells as described (6). In the experiment with 5 μM mitoparaquat, drug or 0.1% DMSO vehicle control was added 1 day post Mtb infection and images acquired after 5 hours incubation. SYTOX^®^ Green Nucleic Acid Stain (Life Technologies) was added to culture medium 30 min before image acquisition. Macrophages were imaged using a Nikon Ti-E inverted microscope fitted with a 20X objective (Nikon, CFI S Plan Fluor 0.45 NA) and 2–5 arbitrary images per well acquired with NIS Elements (Nikon). Cell necrosis was quantified using a previously described method (6).

### Statistical analysis

The following statistical analyses were performed using Prism 7 (GraphPad): two-way ANOVA or one-way ANOVA with Dunn’s or Tukey’s post-test and Fisher’s exact test. Error bars represent the standard error of mean. Post-test *P*-values were defined as follows: Not significant, *P*>0.05; *P<0.05; ** *P*<0.01; ***P<0.001; and ****P<0.0001. The statistical tests used for each figure can be found in the corresponding figure legend. Where the *n* value is given and not represented graphically in the figure, *n* represents the number of zebrafish used for each experimental group.

### Software used

The following software was used: NIS-Elements for image acquisition in wide-field and confocal microscopy, ImageJ (https://fiji.sc/) for image analysis of macrophage death, GraphPad Prism 7.0 (GraphPad Software, Inc., San Diego, CA) for data graphing and statistical analyses, and CorelDRAW (CorelDRAW Graphics Suite x5) for figure preparation.

## Supplementary Material

1
