# Supplementary material for: TNF induces pathogenic mitochondrial ROS in tuberculosis through reverse electron transport
Source: Science. Author manuscript; Available in PMC 2022 Jul 27. (PMC7612974; doi:10.1126/science.abh2841)
Supplement: 1 [file NIHMS1818883-supplement-1.pdf]

Science
AAAS

Supplementary Materials for

TNF induces pathogenic mitochondrial ROS in tuberculosis through reverse electron transport

Francisco J. Roca[1,4], Laura J. Whitworth[1,2], Hiran A. Prag[3], Michael P. Murphy[1,3] and Lalita Ramakrishnan[1,2,*]

Correspondence to: lalitar@mrc-lmb.cam.ac.uk

This PDF file includes:

Figs. S1 to S5
Tables S1 to S2
Captions for Data S1

Other Supplementary Materials for this manuscript include the following:

Data S1 (Excel file)

Figure S1

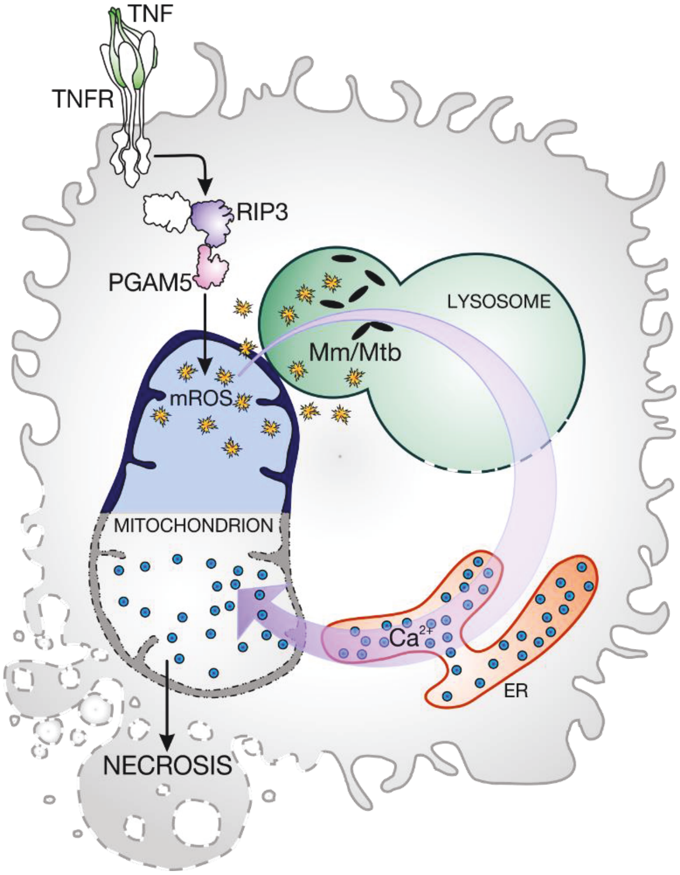

Figure S1.

Excess TNF induces mROS and necrosis of mycobacterium-infected macrophages.

Simplified illustration of the necrosis pathway triggered by excess TNF. Mm, *Mycobacterium marinum*; Mtb, *M. tuberculosis*; ER, endoplasmic reticulum; Ca^{2+}, calcium.

Figure S2

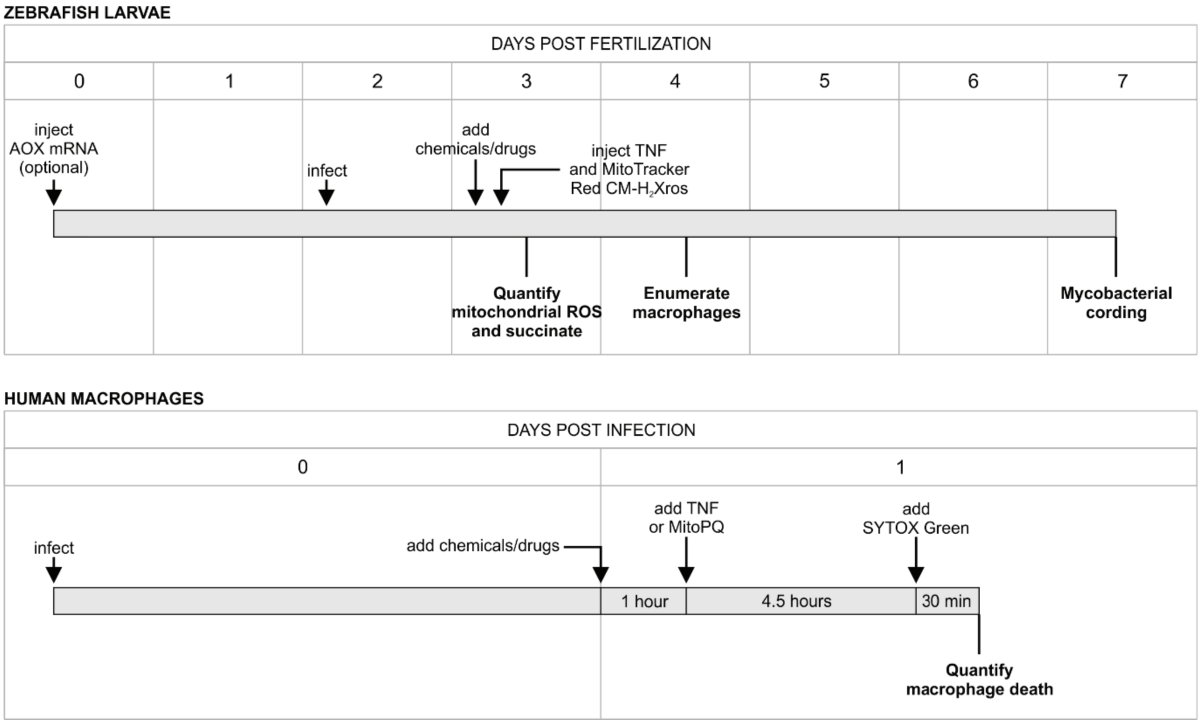

Fig. S2.
Diagram showing the experimental design used in the study in zebrafish larvae and human macrophages.

AOX mRNA, messenger RNA for alternative oxidase from *Ciona intestinalis*; MitoPQ, MitoParaquat. See Materials and Methods for more details about route and time of administration of chemicals, drugs, and TNF in zebrafish larvae.

Figure S3

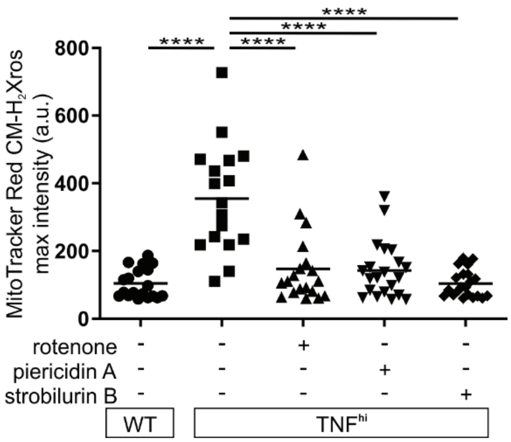

Fig. S3.

Complex I inhibitors with different mechanisms of action reduce mROS in TNF-high conditions.

Quantification of mROS 1 dpi with Mm in larvae that are wild-type (WT) or TNF^{hi} treated with rotenone, piericidin A, strobilurin B, or vehicle. Horizontal bars represent means; ****P<0.0001 (one-way ANOVA with Tukey's post-test). Representative of two independent experiments.

Figure S4

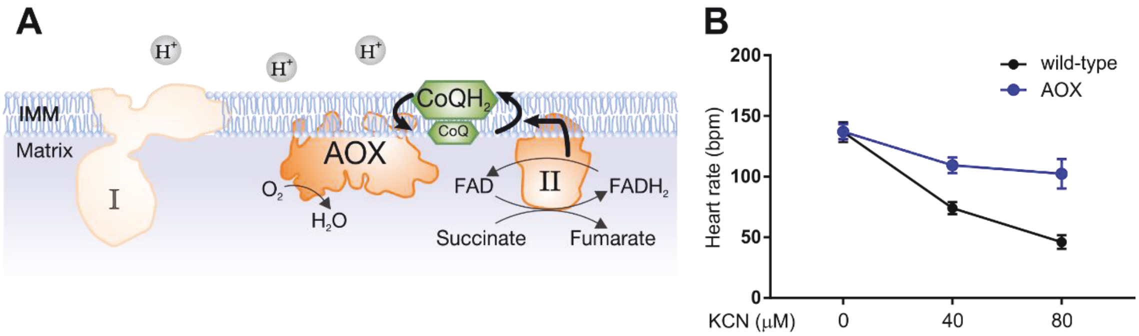

Fig. S4.
AOX-expressing zebrafish larvae are resistant to inhibition of complex IV by cyanide.

(A) Illustration demonstrating how AOX expression decreases the CoQH2 pool and prevents RET mROS production at complex I. Compare with Fig. 2B. AOX, alternative oxidase; IMM, inner mitochondrial membrane; I-II, complexes. (B) Comparison of heart rate (beats per minute) in 3 dpf wild-type or AOX-expressing animals treated with KCN or vehicle. $P<0.0001$ (two-way ANOVA).

Figure S5

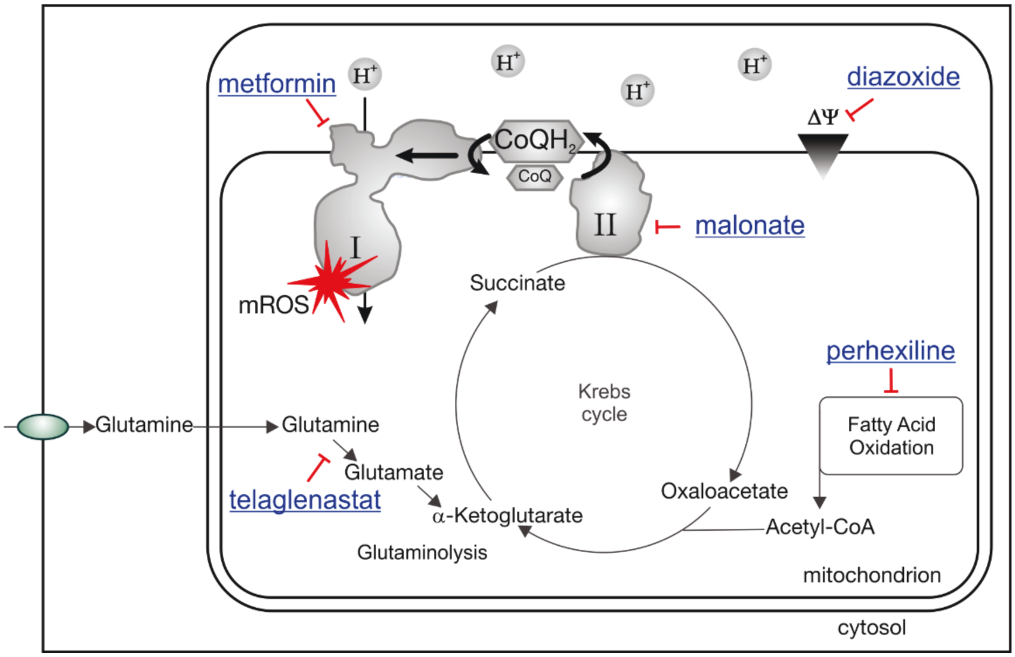

Figure S5.

Currently available drugs can intercept TNF-induced mROS production and inhibit necrosis of mycobacterium-infected macrophages.

Schematic diagram showing the new druggable targets identified in this work to inhibit TNF-elicited RET mROS and necrosis of mycobacterium-infected macrophages. Blue underlined, drugs; red blunted arrows, inhibition.

Compound	Mechanism of action	Human Drug?
**Modulators of CI**		
Rotenone	Inhibitor of complex I (*12, 14*)	No. Plaguicide in agriculture (*39*)
Piericidin A	Inhibitor of complex I (*14*)	No. Laboratory reagent
Strobilurin B	Inhibitor of complex I (*14*)	No. Fungicide in agriculture (*40*)
Metformin	Inhibitor of complex I (*15*)	Yes, oral, anti-diabetic (*41, 42*)
Phenformin	Inhibitor of complex I (*15*)	No. Withdrawn from clinical use (*41*)
**Modulators of CII**		
TTFA (thenoyltrifluoroacetone)	Inhibitor of complex II (*43*)	No. Laboratory reagent
Atpenin A5	Inhibitor of complex II (*44*)	No. Laboratory reagent
DM-malonate (dimethyl malonate) as a source of the inhibitor malonate	Inhibitor of complex II (*19*)	Pro-drug shown to prevent ischemia-reperfusion injury in models of heart attack (*28*)
**Mitochondrial uncouplers**		
FCCP (Carbonyl cyanide-4-(trifluoromethoxy)phenylhydrazone)	Protonophore (*15*)	No. Laboratory reagent
DNP (2.4-dinitrophenol)	Protonophore (*45*)	No. Laboratory reagent
Nigericin	Ionophore, K^+/H^+ exchanger (*15*)	No. Laboratory reagent
Diazoxide	Activator of ATP-sensitive potassium channels (KATP channels) (*46*)	Yes, oral, for hyperinsulinemic hypoglycemia (*47*)
**Modulators of glycolysis**		
UK5099	Inhibitor of the mitochondrial pyruvate carrier (*48*)	No. Laboratory reagent
**Modulators of FAO**		
Perhexiline	Inhibitor of CPT1/2 (*49*)	Yes, oral, antianginal (*50*)
4-BrCA (4-Bromocrotonic acid)	Inhibitor of 3-ketoacyl-CoA thiolase (KAT) (*51*)	No. Laboratory reagent
**Modulators of glutaminolysis**		
GPNA (L-γ-Glutamyl-p-nitroanilide)	Inhibitor of SLC1A5 (Gln transporter) (*52*)	No. Laboratory reagent
BPTES	Inhibitor of glutaminase 1 (GLS1) (*52*)	No. Laboratory reagent
Telaglenastat (CB-839)	Inhibitor of glutaminase 1 (GLS1) (*52*)	Yes, oral, In clinical trials for cancer (*53*)
R-162	Inhibitor of GDH1 (*54*)	No. Laboratory reagent
**TCA intermediates and modulators of TCA**		
Methyl pyruvate (M-pyruvate)	Cell permeable source of pyruvate (*55*)	No. Laboratory reagent
Diethyl succinate	Cell permeable source of succinate (*19*)	No. Laboratory reagent
DEBM (diethyl butyl malonate)	Inhibitor of the mitochondrial succinate/malate antiporter (*19*)	No. Laboratory reagent

Dimethyl glutamate (DM-glutamate)	Cell permeable source of glutamate (*56*)	No. Laboratory reagent
**Others**		
MitoParaquat (MitoPQ)	Mitochondria-targeted redox cycler that produces superoxide by redox cycling at the flavin site of complex I (*57*)	No. Paraquat used as herbicide in agriculture (*58*)

Table S1.
Small molecules used in the study.

Compound	Concentration used (tested)	Toxic effects observed
Rotenone	6.25 (6.25-100) nM	Death 24 hours post administration with concentrations > 12.5 nM
Piericidin A	50 (5-500) nM	Necrotic tissues 24 hours post administration with concentrations > 50 nM
Strobilurin B	100 (5-500) nM	Slow heart rate 24 hours post administration with concentrations > 100 nM
Metformin	20 (1-40) μM	Death 24 hours post administration with 40 μM. No toxic effects observed with 20 μM over 4 days
Phenformin	20 (1-40) μM	Gray yolk 24 hours post administration with 40 μM. No toxic effects observed with 20 μM over 4 days
TTFA	1 (0.25-20) μM	Death 24 hours post administration with 5 μM. Curved spines in some larvae 24 hours post administration with 1 μM
Atpenin A5	2.5 (2.5-1000) nM	Necrotic tissues 24 hours post administration with concentrations >2.5 nM and up to 25 nM. Death 4 hours post administration with concentrations >25 nM
dimethyl malonate	10 (1-100) μM	No toxic effects observed for any of the concentrations tested 24 hours post administration. No toxic effects observed with 10 μM over 4 days (not tested for other concentrations)
FCCP	50 (50-500) nM	Necrotic tissues observed in 2 hours with 200 nM. Necrotic tissues 24 hours post administration with 50 nM
2.4-dinitrophenol	100 (10-1000) nM	Necrotic tissues 24 hours post administration with concentrations >500 nM
Nigericin	5 (0.05-5) μM	Death 24 hours post administration with 5 μM
Diazoxide	50 (12.5-2500) nM	No toxic effects observed for any of the concentrations tested 24 hours post administration. No toxic effects observed with 50 nM over a period of 4 days (not tested for other concentrations)
UK5099	10 (1-50) μM	Gray yolk and edema 24 hours post administration with concentrations >10 μM
Perhexiline	10 (0.01-10) μM	No toxic effects observed for the concentrations tested over 4 days
4-Bromocrotonic acid	10 (1-20) μM	No toxic effects observed for the concentrations tested 24 hours post administration
GPNA	10 (0.1-100) μM	No toxic effects observed for the concentrations tested 24 hours post administration
BPTES	5 (2-5) μM	No toxic effects observed for the concentrations tested over 4 days
Telaglenastat	5 (0.5-5) μM	No toxic effects observed for the concentrations tested over 4 days
R-162	1 (0.1-1) μM	No toxic effects observed for the concentrations tested over 4 days

Table S2.
Toxic effects observed in zebrafish larvae after pharmacological interventions.

Data S1. (separate Excel file)
Raw data and summary of the analysis for the experiments showed in Fig. 5, A and B.

Data S1

	uninfected-WT	Mm-WT	uninfected-TNF	Mm-TNF^hi	Mm-TNF^hi-GPNA	Mm-TNF^hi-BPTES
EXPERIMENT 1	10.19964	12.5342	11.56649	15.87831	8.191469	9.621429
	10.25871	10.9107	8.89771	12.96664	9.186496	9.006775
	12.23304	13.3213	9.377055	11.00655	9.754825	9.513851
	10.55048	11.1403	10.29649	12.59976	9.928756	7.913414
	10.75182	10.9969	8.885279	11.56042	10.32894	8.876832
EXPERIMENT 2	8.266625	9.94793	8.52337	11.64261	8.416775	11.22308
	8.31334	9.34203	9.81538	10.46372	7.693635	9.48613
	8.65511	9.42176	9.01085	10.15769	8.581375	11.09407
	7.471995	9.06886	8.863855	9.744265		12.00439
	10.55495	8.39195	9.79467	12.70169		
	8.844005	9.2075	8.69321	13.26537		
EXPERIMENT 3	9.669373	11.9157	10.60354	11.69141	10.28906	9.950385
	9.28354	9.16828	9.64768	10.132	9.7715	11.43472
	9.953755	13.3317	9.425665	11.85374	10.10445	8.064325
	9.63323	10.9463	8.93133	12.69284	10.53676	11.23378
	9.52803	9.16405	9.34577	13.90738		
	10.30629	9.90041	7.994845	12.18015		

	EXPERIMENT 1	EXPERIM	EXPERIMENT 3	COMBINED (with pooled SD)
**uninfected-WT**				
N	5	6	6	17
mean	10.8	8.68	9.73	9.674117647
SD	0.832	1.03	0.356	0.732714286
**Mm-WT**				
N	5	6	6	17
mean	11.78	9.23	10.74	10.51294118
SD	1.087	0.5086	1.661	1.085428571
**uninfected-TNF**				
N	5	6	6	17
mean	9.805	9.117	9.325	9.392764706
SD	1.14	0.5576	0.8572	0.831
**Mm-TNF^hi**				
N	5	6	6	17
mean	12.8	11.33	12.08	12.02705882
SD	1.891	1.44	1.243	1.4985
**Mm-TNF^hi-GPNA**				
N	5	3	4	12
mean	9.478	8.231	10.18	9.40025
SD	0.8283	0.4722	0.3223	0.5805
**Mm-TNF^hi-BPTES**				
N	5	4	4	13
mean	8.986	10.95	10.17	9.954615385
SD	0.6792	1.057	1.551	1.05408

	uninfected-WT-TNF^{hi}	Mm-WT-TNF^{hi}	Mm-RIP3 mo-TNF^{hi}	Mm-PGAM5 mo-TNF^{hi}
**EXPERIMENT 1**	8.32489811	9.4561594	7.75383391	8.53134
	9.220129	10.1714924	7.62524815	7.60957
	8.0947494	9.222644	8.492279	7.42046
	8.0255603	10.757272	8.42630054	8.83444
	7.4381754	9.78669832	8.33939946	8.18587
	7.42469075	11.0531316	8.84089999	8.49575
**EXPERIMENT 2**	7.42881553	11.6159017	9.1216251	8.45264
	8.48136285	9.9865356	8.5747611	8.54323
	8.15280383	10.7075206	9.0975498	8.12821
	8.39264242	10.9007516	8.9212862	8.49751
	7.62696943	11.5066377	7.7993937	7.9872
			8.6476334	8.99449

	EXPERIMENT 1	EXPERIMENT 2	COMBINED (with pooled SD)
**uninfected-WT-TNF^{hi}**			
N	6	5	11
mean	8.088	8.017	8.055727273
SD	0.6639	0.4672	0.576477778
**Mm-WT-TNF^{hi}**			
N	6	5	11
mean	10.07	10.94	10.46545455
SD	0.7244	0.66	0.695777778
**Mm-RIP3 mo-TNF^{hi}**			
N	6	6	12
mean	8.246	8.694	8.47
SD	0.4655	0.4928	0.47915
**Mm-PGAM5 mo-TNF^{hi}**			
N	6	6	12
mean	8.18	8.434	8.307
SD	0.5575	0.3532	0.45535
